# Changing nurses’ views of the therapeutic environment: randomised controlled trial

**DOI:** 10.1192/bjo.2018.87

**Published:** 2019-01-29

**Authors:** Emese Csipke, Til Wykes, Stephen Nash, Paul Williams, Leo Koeser, Paul McCrone, Diana Rose, Tom Craig

**Affiliations:** Doctor, Senior Research Fellow, Division of Psychiatry, University College London, UK; Professor Dame, Institute of Psychiatry, Psychology and Neuroscience, King's College London, South London and Maudsley NHS Foundation Trust, UK; Statistician, London School of Hygiene and Tropical Medicine, UK; Statistician, Institute of Psychiatry, Psychology and Neuroscience, Kings College London, UK; Research Assistant, Institute of Psychiatry, Psychology and Neuroscience, Kings College London, UK; Professor of Health Economics, Institute of Psychiatry, Psychology and Neuroscience, King's College London, UK; Professor of User-Led Research, Institute of Psychiatry, Psychology and Neuroscience, King's College London, UK; Professor of Social Psychiatry, Institute of Psychiatry, Psychology and Neuroscience, King's College London, UK

**Keywords:** Nursing, in-patient, therapeutic, evidence based, psychological

## Abstract

**Background:**

Although patients value evidence-based therapeutic activities, little is known about nurses' perceptions.

**Aims:**

To investigate whether implementing an activities training programme would positively alter staff perceptions of the ward or be detrimental through the increased workload (trial registration: ISRCTN 06545047).

**Method:**

We conducted a stepped wedge cluster randomised trial involving 16 wards with psychology-led nurse training as the intervention. The main outcome was a staff self-report measure of perceptions of the ward (VOTE) and secondary outcomes measuring potential deterioration were the Index of Work Satisfaction (IWS) and the Maslach Burnout Inventory (MBI). Data were analysed using mixed-effects regression models, with repeated assessments from staff over time.

**Results:**

There were 1075 valid outcome measurements from 539 nursing staff. VOTE scores did not change over time (standardised effect size 0.04, 95% CI –0.09 to 0.18, *P* = 0.54), neither did IWS or MBI scores (IWS, standardised effect size 0.02, 95% CI –0.11 to 0.16, *P* = 0.74; MBI standardised effect size –0.09, 95% CI –0.24 to 0.06, *P* = 0.24). There was a mean increase of 1.5 activities per ward (95% CI –0.4 to 3.4, *P* = 0.12) and on average 6.3 more patients attended groups (95% CI –4.1 to 16.6, *P* = 0.23) following training. Staff feedback on training was positive.

**Conclusions:**

Our training programme did not change nurses' perceptions of the ward, job satisfaction or burnout. During the study period many service changes occurred, most having a negative impact through increased pressure on staffing, patient mix and management so it is perhaps unsurprising that we found no benefits or reduction in staff skill.

**Declaration of interest:**

None.

## Introduction

As bed numbers continue to fall, mental health wards are reserved for the most acutely ill with consequent increases in the proportion of in-patients who are compulsorily detained. These factors are likely to increase levels of behavioural disturbance.^1^^,^^2^ This can lead to a very fraught ward atmosphere, with nurses spending the majority of their time dealing with crises rather than engaging in therapeutic activities or interacting with patients. Long before the Francis report,^2^^,^^3^ the UK Department of Health^4^ acknowledged that in spite of advocating therapeutic environments, this fire-fighting activity, along with an abundance of administrative work, lack of support and inadequate supervision make in-patient wards very challenging for staff. Patients have been reported to spend as little as 4% of their time interacting with nursing staff.^5^ Taken together this often brings about ward environments that appear to be more custodial than therapeutic.

## Nurses and therapeutic care

Nurses report the primary reason for not spending time on therapeutic activities or direct patient contact is the need to focus on resolving crises for a small number of patients, in addition to increased administrative duties.^5^^–^^7^ Yet patients value time spent interacting with nursing staff and taking part in therapeutics activities, regardless of how acutely ill they might be^8^^,^^9^ and such provision improves the patient experience especially for those compulsorily admitted to in-patient wards.^8^^,^^10^ Nurses strive to provide good-quality patient care^11^ and undoubtedly any lack of achievement has an impact on staff morale and burnout,^12^ which has been linked to concerns about recruitment, retention and training of mental health practitioners around the world (see for example Paris & Hoge^13^).

Improved patient-reported quality of care and decreases in staff sicknesses have been reported when staff spent more time in individualised activities^14^ and there is evidence that increases in group activities is related to significantly greater involvement of patients in their own care, greater support between staff and patients, and more opportunities to practise skills needed following discharge.^15^ More recently^6^ it has been advocated that nursing staff should provide evidence-based psychotherapy in in-patient settings and it has been report that patients appreciated activity-based and psychoeducational groups run by a range of health professionals.^16^^,^^17^

Advocacy groups across the world (for example the Mental Health Council of Australia, the National Alliance on Mental Illness) support the implementation of evidence-based therapeutic activities in in-patient settings. However, we are aware that asking nurses to spend more time doing these activities, without a decrease in their other duties, as well as a shrinking number of staff, may lead to negative effects because of a perceived increase in workload.

In the UK many training/accreditation programmes address the issue of the quality of in-patient care such as Starwards and Aiquip (see for example: Bowers *et al*^5^ and Royal College of Psychiatrists^18^). Clarke^19^ has outlined that these programmes could be improved by providing adequate training and supervision for ward-based staff, a suggestion echoed by a US committee dedicated to the welfare of US-based mental health professionals.^20^ All this evidence suggests that activities – especially those that are evidenced based – are beneficial to patients but there has been little information on their impact on the morale and job satisfaction of nursing staff. Most studies are short term and therefore do not investigate whether there are longer-term implications for the sustainability of any changes in nursing staff time allocation.

Our project implemented a programme of evidence-based activities to improve in-patient services and rigorously evaluated their impact. The aims of the study are: (a) to investigate the changes to staff perceptions of the environmental milieu of providing staff training for therapeutic activities and (b) to explore any development of negative effects (particularly on staff morale).

## Method

### Design

The study was a cluster randomised trial of 16 wards (clusters) in a stepped wedge design (whereby the timing of the intervention to a ward is randomised) (trial registration: ISRCTN 06545047, http://www.perceive.iop.kcl.ac.uk/). As all clusters eventually receive the intervention, this design is often used in situations where for ethical reasons the researchers do not wish to deny the intervention to any particular cluster.^21^ Another key reason for selecting this design method was that it allows multiple clusters to be included even when it is not possible to intervene in all clusters simultaneously. The use of this design is relatively uncommon and analysis methods are recent.^22^ For pragmatic reasons the randomisation was carried out in three waves, with the first eight wards being randomised, four in the second wave with the final four following. Further details are described in our companion paper relating to patient experience.^10^

Nursing staff working on these wards during any assessment period were recruited and although they were aware of whether their ward had been randomised or not, assessments took place before any training was completed or activities were run. All staff assessments were self-report. We collected data at nine time points, approximately 6 months apart. Staff completed assessments each time they were available and consented to do so and so repeat measurements per person were permitted.

### The setting

This study was carried out in two mental health trusts that cover five distinct geographic areas representing varied deprivation scores from the high deprivation inner city through to suburban affluent areas (for more detail see Csipke *et al*^8^).

- Borough 1 serves an inner-city population with a high deprivation index. Five 18-bedded wards participated.
- Borough 2 serves a suburban affluent area. Three wards participated, two wards had 22 beds and the remaining one had only 8 beds.
- Borough 3 has a high deprivation score and four 18-bedded wards provide acute in-patient care.
- Borough 4 had two 18-bedded mixed-gender wards that serve an area with a high deprivation score.
- Borough 5 had two 18-bedded wards that serve an area that is more suburban and affluent.

### Participants

Nurses of any grade were eligible to take part. Temporary staff were required to have completed seven shifts in the previous month in order to be eligible so that we could be sure that they were sufficiently familiar with the ward environment to complete the measures. Ethical approval for the study was granted by Bexley and Greenwich Research Ethics Committee (Ref: 07/H0809/49) and all eligible participants were approached and gave written informed consent.

### Main outcome

For all measures obtained by questionnaires it was a requirement that more than 80% of the questions were completed. Questionnaires with less than 80% item completion were excluded as missing.

We used the Views of the therapeutic Environment (VOTE),^11^ as the main outcome. This is a 20-item measure, each on a six-point Likert scale with good reliability and validity that captures staff perceptions of the daily pressures of working on acute in-patient mental health wards. The outcomes investigated were the total scores and for secondary analyses three subscales (workload intensity, team dynamics and interaction anxiety). Low scores represent positive views. VOTE was user developed by nurse researchers with in-patient ward nursing staff specifically for this study to reflect the concerns and views of the nurses. As far as we know there is no other nurse-developed measure of nurse views.

### Secondary outcomes

Our secondary outcomes were as follows.

- The Maslach Burnout Inventory-Human Services Survey (MBI).^23^ This captures work-related ‘burnout’ over 22 items, each on a six-point Likert scale. It has good psychometric properties and is widely used. The MBI consists of three subscales: emotional exhaustion, depersonalisation and personal accomplishment. High scores on the emotional exhaustion and depersonalisation subscales indicate poor functioning but higher scores on the personal accomplishment subscale indicate better functioning
- The Index of Work Satisfaction (IWS).^24^ This consists of 44 items measured on a seven-point Likert scale with a total score reflecting job satisfaction, with higher scores representing less job satisfaction.
- Demographic information on staff. Participants' age, gender, ethnicity, employment band and length of employment were collected.
- Costs. In order to estimate the cost of the intervention we recorded the number of participants in the training sessions and their duration along with the staff input required to deliver the training.

### Post-training feedback

One group session and four interviews were carried out at the end of the trial. Topic guides were developed and nurses were invited to discuss their experience of completing the training programme and running the subsequent groups. In particular, they were asked to reflect on what worked well and what did not. The responses and discussions were recorded and transcribed to provide an additional source of data to compliment results from the quantitative analyses, and to continue to seek nurses' views in a non-quantitative manner.

### Staff training intervention

In keeping with the ethos of involving ward nurses at every stage of the study, the Trust clinical leads, nursing management and direct care staff on the wards met several times to consult on the evidence-based activities that were eventually chosen. National Institute for Health and Care Excellence (NICE) guidelines were taken into account, and the group chose to include other staff (occupational therapists and pharmacists) in the training as they also have a presence on the ward. This consultancy group decided to have four activities as compulsory as they applied to all wards.

- A single session of cognitive–behavioural therapy-based communications and understanding/avoiding aggression training for nurses (cofacilitated by a patient educator).
- Social cognition and interaction training^25^ aimed at helping people understand social situations better in order to avoid misunderstandings, a common occurrence on wards.
- Computerised cognitive remediation therapy (in order to involve occupational therapists), designed to address cognitive deficits such as problems with memory, organisation and concentration.^26^
- Where pharmacists were available, they were recruited to run a medication education group.

Two optional nurse-provided therapies were chosen by wards themselves based on their patient's needs (wards had unique mixes of patients) and the mix of skills available from the following: problem-solving skills, emotional coping skills group, hearing voices group, relaxation/sleep hygiene and coping with stigma group.

Training for each of the activities lasted 2–3 h. Clinical psychologists delivered the training, and cofacilitated the groups themselves alongside the nurses until the nurses were able and confident to deliver it independently. Nurses were required to run the groups independently by the end of 6 months. The groups themselves ran for 45 min once a week. See http://www.perceive.iop.kcl.ac.uk/ and Csipke *et al*^8^ for more detailed information.

### Randomisation

The training intervention time was randomised by one of the trial statisticians using the random permutations of numbers using the *ralloc* procedure in Stata. Wards 1 to 8 were randomised first (two wards at a time), then wards 9 to 12, and finally wards 13 to 16. After the baseline period two wards were selected at random to receive staff training, with a further two wards selected every 6 months until all wards had received the training. The final four wards joined the study at a later period as they became available to participate in the study, therefore it was not possible to collect more extensive preintervention data. The design is shown in Table 1.

**Table 1:** Randomisation schedule^a^

Borough		Time – start of data collection period								
		1 November 2008	1 June 2009	1 January 2010	1 June 2010	1 January 2011	1 June 2011	1 January 2012	1 June 2012	1 January 2013
	Ward	1	2	3	4	5	6	7	8	9
1	1	0	1	1	1	1	–	–	–	–
2	2	0	1	1	1	1	–	–	–	–
2	3	0	0	1	1	1	–	–	–	–
3	4	0	0	1	1	1	–	–	–	–
1	5	0	0	0	1	1	–	–	–	–
2	6	0	0	0	1	1	–	–	–	–
1	7	0	0	0	0	1	–	–	–	–
1	8	0	0	0	0	1	–	–	–	–
3	9	0	0	0	0	0	1	1	–	–
3	10	0	0	0	0	0	1	1	–	–
3	11	0	0	0	0	0	0	1	–	–
3	12	0	0	0	0	0	0	1	–	–
4	13	–	–	–	–	–	–	0	1	1
4	14	–	–	–	–	–	–	0	1	1
5	15	–	–	–	–	–	–	0	0	1
5	16	–	–	–	–	–	–	0	0	1

a. 0, represents data collection prior to receipt of the staff training (i.e. control wards); 1, represents data collection after receipt of the staff training (i.e. intervention wards). Time is measured in periods of 6 months. Further information available from the authors on request.

### Statistical analysis

The effect of staff training was estimated through effects on staff members including more general ward effects as well as individual-level data. All analysis was carried out using Stata version 11 or later.

The primary outcome (VOTE score) was analysed using a mixed-effects regression model with a random effect to account for the variance because of repeated measurements within staff. Ward and time were accounted for as fixed effects, by using an indicator variable to account for the ward and a time variable that counted collection periods as shown in Table 1. A similar method was used in the service arm of the study^10^ but without the repeated measures random effect.

The analysis of the VOTE subscales (workload intensity, team dynamics and interaction anxiety) and secondary outcomes followed the same pattern as for the main analysis. The following variables derived from previous studies^6^^,^^9^ were considered as potential confounders: gender, age, ethnicity, employment band, first language and length of employment. Confounding factors were assessed and included in the model if they were associated with both the intervention and the outcome with a significance of *P*<0.10. We performed exploratory analyses by including an interaction between the subgroup and the staff training arm and tested the nested models using likelihood-ratio tests on all outcomes to explore potential differential effects between men and women, and between White and Black and minority ethnic staff.

## Results

Staff members could participate on more than one occasion. In total, 560 staff members consented to take part, with 444 taking part in the pretraining intervention and 280 post-training, of whom 64% were women. We had 1075 valid VOTE scores (677 for the pretraining intervention and 398 post training) from 539 staff members. Only 3.4% of questionnaires were excluded as a result of completing less than 80% of the items.

Demographic characteristics between the pre- and post-training phases of the trial were balanced. Table 2 describes characteristics of participants as a single group at first point of entry to the study. In the UK, Bands 2–6 are direct care staff working with patients on the wards, with Band 2 nurses being student nurses and Band 6 nurses being senior direct care nurses. Bands 7 and 8 are managerial nurses.

**Table 2:** Baseline characteristics of staff participants

Characteristic	Pre-training wards	Post-training wards
Overall trial population, *n*	444	280
Gender, *n* (%)	439	278
Men	171 (39.0)	98 (35.3)
Women	268 (61.0)	180 (64.7)
Age,^a^ mean (s.d.)	36.9 (10.1)	38.2 (10.7)
Ethnicity, *n* (%)	434	274
White	167 (38.5)	114 (41.6)
Mixed	14 (3.2)	5 (1.8)
Asian	32 (7.4)	18 (6.6)
Black	208 (47.9)	126 (46.0)
Other	13 (3.0)	11 (4.0)
First language,	427	267
English	258 (60.4)	167 (62.5)
Not English	169 (39.6)	100 (37.5)
Band	414	261
2	33 (8.0)	20 (7.7)
3	113 (27.3)	69 (26.4)
4	3 (0.8)	1 (0.4)
5	184 (44.4)	117 (44.8)
6	59 (14.3)	37 (14.2)
7	21 (5.1)	15 (5.8)
8	1 (0.2)	2 (0.8)
Length of employment on ward (months),^a^ mean (s.d.)	44.1 (63.6)	55.6 (75.1)

a. For staff with repeated measures, the earliest response was used.

### Wards

Demographic characteristics of baselines characteristics broken down by wards are presented in supplementary Table 1 (available at https://doi.org/10.1192/bjo.2018.87). The number of completed assessments per time point per ward are included in supplementary Table 2. We did not include an interaction with time but site effects are listed in supplementary Table 3.

### Outcomes

#### What effects did activities have on staff views of the wards?

In total, 677 VOTE scores were from 428 individuals on pretraining wards (mean score 68.2, s.d. = 12.0) and 398 scores were from 271 individuals on post-training wards (mean score 70.3, s.d. = 12.7), where a lower score is indicative of a better perception of the ward. Results from the regression model showed no evidence of a change in VOTE scores (standardised effect size (ES) = 0.04, 95% CI –0.09 to 0.18, *P* = 0.54) (Table 3).

**Table 3:** Adjusted results for Views of the therapeutic Environment (VOTE) and VOTE subscales (standardised scales)

	Training intervention, effect size	95% CI	s.e.	*P*	Observations, (staff). *n*	ICC
VOTE	0.04	(–0.09 to 0.18)	0.07	0.54	1075 (546)	0.61
VOTE subscales						
Workload intensity^a^	0.02	(–0.13 to 0.18)	0.08	0.78	1076 (526)	0.47
Team dynamics^b^	0.08	(–0.08 to 0.23)	0.08	0.34	996 (488)	0.52
Interaction anxiety^b^	0.001	(–0.15 to 0.15)	0.08	0.99	996 (488)	0.59

s.e. standard error; ICC, intraclass coefficient.

a Adjusted for length of employment.

b Adjusted for age.

Our exploratory analysis examined effects for staff subgroups and suggested a difference between men and women but not for ethnicity. The model including an interaction between trial arm and gender estimated a negative effect for men (ES = 0.19, 95% CI 0.01 to 0.36, *P* = 0.02) and a non-significant benefit for women (ES = –0.05, 95% CI –0.20 to 0.11, *P* = 0.55).

A clinically meaningful increase in a standardised effect is difficult to ascertain in this new measure, VOTE. As a guide, when using Cohen's *d* (a different standardised effect measurement), the Food and Drug Administration of the USA offers the guideline that an effect is large if *d* > 0.8 and small if *d* < 0.5.

Our interview and focus group data from nurses suggested that they enjoyed running the activities and appreciated the opportunity to feel that they were providing something therapeutic rather than paperwork and crisis management. But they also reported that it was hard to be consistent, especially when there were high numbers of temporary staff on the ward. Activities were repetitive and they and patients sometimes became bored of them especially when they were in hospital for longer periods.

### Secondary outcome analyses

Secondary analyses on satisfaction and burnout show no significant effects as for the main outcomes (Table 4). For the factors in the MBI we compared the factors with the norms for mental health staff in the MBI Handbook.^23^ We found that emotional exhaustion was higher both before and after training (pretraining 21.5 (s.d. = 11.1) post-training, 22.3 (s.d. = 12.1 compared with the MBI handbook, 16.89 (s.d. = 8.90)). Depersonalisation was about the same but personal accomplishment was higher (i.e. better) (pretraining 35.5 (s.d. = 6.1) post-training, 35.2 (s.d. = 6.0) compared with the MBI Handbook 30.87 (s.d. = 6.37). But no factors were affected by the training.

**Table 4:** Adjusted results for Index of Work Satisfaction (IWS) and Maslach Burnout Inventory (MBI)

	Pre-training, mean (s.d.)	Post-training, mean (s.d.)	Training effect, effect size (95% CI)	*P*	Observations (staff), *n*	ICC
IWS	160.4 (30.5)	165.3 (29.8)	0.02 (–0.11 to 0.16)	0.74	1028 (523)	0.67
MBI^a^	39.5 (16.4)	40.7 (17.7)	−0.09 (–0.24 to 0.06)	0.24	901 (457)	0.63
MBI subscales						
Emotional exhaustion^a^	21.5 (11.1)	22.3 (12.1)	−0.11 (–0.26 to 0.04)	0.16	930 (470)	0.58
Depersonalisation^a^	5.8 (5.0)	5.8 (4.9)	−0.02 (–0.19 to 0.15)	0.83	924 (464)	0.49
Personal accomplishment	35.5 (6.1)	35.2 (6.0)	0.03 (–0.14 to 0.19)	0.73	1012 (521)	0.44

ICC, intraclass coefficient.

a Adjusted for age.

### Did we increase the number of ward activities?

From data obtained from ward records, the mean number of activities per week before the staff training was 5.9 whereas after training it was 8. The regression analysis yielded a mean increase of 1.5 activities following the training (95% CI –0.4 to 3.4, *P* = 0.12) (Table 5). Before and after the training the average number of patients in activities was 28 and 39, respectively. A regression model estimated an average increase of 6.3 patients attending groups following the training (95% CI –4.1 to 16.6, *P* = 0.23).

**Table 5:** Ward reported activities (adjusting for ward effects)

Outcome	Average training effect	95% CI	standard error	*P*
Number of activities provided	1.48	(–0.40 to 3.37)	0.94	0.12
Total number of participants	6.27	(–4.05 to 16.59)	5.10	0.23

### What was the cost of the intervention?

Over the 16 wards in this study, we estimated that the total cost of staff training amounted to approximately £156 000. Taking into account the average occupancy rates and the average post-training follow-up of 55 weeks this yielded a cost of £10 per patient-week.

## Discussion

Our study sought to investigate changes in perceptions of the ward environment among those working on acute in-patient mental health wards by training staff in the skills needed to provide evidence-based therapeutic activities. We also sought to explore the development of any negative impact on staff morale. Our study was unique in that the main outcome measure, as well as the development of the training programme, involved the nurses working on the very wards included in the study in order to investigate what it is that matters to them rather than researcher-imposed priorities. Over the course of the study, nurses' perceptions of the wards did not improve. Nevertheless, we did make an impact on the day-to-day life of the ward in a number of ways, which needs to be balanced against the positive effects noted in patients.^8^

### Impact of training on nurses

There was no strong evidence for change in nurses' perceptions of the ward milieu 6 months following the delivery of the training. Our study was run in an environment that was already in flux, which makes attempting to change systems a challenge. We did find that workload intensity and overall VOTE scores significantly worsened (in men) following training. As we carried out several analyses this result is not conclusive but is interesting. We consider that the main result of no effect, and that our training was clearly valued, could be a reflection of the changes in the local National Health Service (NHS) services during the study although we have no empirical support for this contention. Three of the five catchment areas in our study reduced the number of wards, and budgets were also reduced. In one ward, staff had to reapply for their own jobs, and some wards did not have ward managers but were run by junior staff or overseen on a part-time basis only. In light of this turbulent background it is surprising that being asked to deliver more activities had no effect on their view of the ward.

In spite of the training requiring nurses to engage and interact with patients more frequently, there were no effects on interaction anxiety, as measured by the VOTE subscale. In the post-training feedback we found that nurses reported their confidence was growing following training. For instance:

‘Because we were a bit unsure of ourselves…But I think once we got into it we didn't have a problem at all, we got more and more confident and it's not a problem anymore to, and I think it's helped us to run other groups because we're more confident in that so now we can run other groups as well without any problem.’

We did not find any differences in burnout, as measured by the MBI. Emotional exhaustion was higher in our sample both pre- and post-training than the means reported in the manual, which may reflect the turbulent nature of the wards. However, it is interesting that there was no effect on personal accomplishment (although this was near ceiling pre-training; meaning that the scale range allowed little scope to detect improvements post-training). There were also no effects on the MBI depersonalisation scale nor on the interaction anxiety measure of VOTE, so increases in activities did not have either a beneficial or a detrimental effect. Even though we invested on wards and observed positive effects on patients who were admitted under legal sanction, neither voluntarily admitted patients or nursing staff demonstrated any change of view. For female members of staff being part of the training group seemed to mitigate time effects (the changes wards underwent) but not for men. In support of the lack of changes in views of the ward, our measure of job satisfaction did not change either as a result of the training intervention.

### Impact on wards

Our intervention consisted of training nurses to deliver evidence-based activities with more talking therapies for example hearing voices groups as recommended by NICE.^27^ We had no hypotheses relating to the increase in activities, but nevertheless thought it worth looking at. We had no control over the number of sessions that were actually run or how many patients participated; however, we were successful in increasing the average number of activities albeit this was not statistically significant. All wards had an activity schedule at baseline and it is not clear how many of these activities were substituted by the therapeutic ones that training was provided for in this study. However, the activities that the training was provided for were evidence-based talking therapies.^27^

The cost of the training was small, and we have reported that the increase in spending per patient was negligible. Patients also reported that they had more contacts and activities following the skills training, which patients said they valued. In our companion paper we considered whether the training might have any knock-on effects on the cost of service use as reported by patients but no statistically significant effect was found.^9^ Administrative duties and crisis management often means that therapeutic activities are relegated to the bottom of the ward's to-do list.^6^^,^^7^

### Costs and cost-effectiveness

Training required investment and when apportioned over the wards and patients this amounted to £10 per patient-week. The cost of meaningful service contacts and activities increased (non-significantly) by £33 per patient-week following training (including the training cost). This does not reflect an actual increase in expenditure but rather a relative increase in resources from a fixed amount. For example, if the actual cost per patient-week of an in-patient stay is £2100 (7 days at £300 per day) then £33 per week extra represents a shift of 2% towards direct patient care.

### Clinical implications

One of the most common complaints about in-patient services is the extreme boredom and lack of therapeutic activities occurring on the wards (see for example Care Quality Commission^28^ and Walsh & Boyle^29^). There was a slight increase in the number of activities and we had positive feedback from those patients participating in the study,^8^ demonstrating that with effort and dedication wards can be changed. Although there was variation in terms of implementation success it should be possible to adopt our design to increase the skills and confidence of nursing staff, which has often been found to be perceived as poor.^3^ This alone would be a positive outcome, not well captured in our current study but revealed in feedback from staff.

### Strengths and limitations

The generalisability of findings is fundamental in research. The original Trust taking part in the project is part of a longstanding and nationally recognised academic NHS partnership and has recently become part of an academic science centre. The users of these services are research active, as are the staff working in them, and senior management are very supportive of research activities. However, we also tested the effects in a trust with fewer academic ties, although we did not perform any statistical tests on trusts (or boroughs) because of multiplicity and absence of any original hypothesis. We believe our results are generalisable to comparable trusts in large cities nationwide regardless of their research activity. We also gave the nursing staff a choice about which optional groups they could run based on their own perceived needs. Future research could investigate the differences between qualified and unqualified staff, especially as it is often unqualified staff who spend the most face-to-face time with patients.

The stepped wedge design did lead to some challenges not least because of changes (for example managerial and staffing levels) to wards during the study so potentially increasing variation, a problem in all pragmatic health service studies. But this design also had benefits over a simple cross-sectional comparison. Neither the wards nor the researchers knew which wards would be randomised next. Pre-intervention measures of the variables that were to be our key outcomes were measured by self-report prior to each randomisation so neither staff nor patients in the control wards knew whether they would receive the active treatment. We followed CONSORT guidelines and the CONSORT checklist for the study can be found in supplementary Table 4.

Some practicalities of implementing the study could not be avoided. Three different psychologists were employed to carry out the training of ward staff, which may have led to training variation although the study team thought that quality improved as more experience was gained over time.

## Implications

Implementing staff training in an unstable environment had many challenges. Nurses are working in a pressured system and their perceptions were mixed in spite of the positive views of our project. The number of activities taking place shifted in the right direction and were a clear benefit of the training. Our investigation of the effects on patients showed that we did benefit those individuals who have been the most critical – individuals who are compelled to receive treatment under a legal sanction.^8^ This was achieved without having a large detrimental effect on staff even if we did not achieve our goal of improving their perceptions of the working environment.

## Supplementary material

For supplementary material accompanying this paper visit http://dx.doi.org/10.1192/bjo.2018.87. click here to view supplementary material
